# What You Find Depends on How You Measure It: Reactivity of Response Scales Measuring Predecisional Information Distortion in Medical Diagnosis

**DOI:** 10.1371/journal.pone.0162562

**Published:** 2016-09-14

**Authors:** Martine Nurek, Olga Kostopoulou

**Affiliations:** 1 Department of Primary Care & Public Health Sciences, Faculty of Life Sciences & Medicine, King’s College London, London, United Kingdom; 2 Department of Surgery & Cancer, Faculty of Medicine, Imperial College London, London, United Kingdom; Technion Israel Institute of Technology, ISRAEL

## Abstract

“Predecisional information distortion” occurs when decision makers evaluate new information in a way that is biased towards their leading option. The phenomenon is well established, as is the method typically used to measure it, termed “stepwise evolution of preference” (SEP). An inadequacy of this method has recently come to the fore: it measures distortion as the total advantage afforded a leading option over its competitor, and therefore it cannot differentiate between distortion to strengthen a leading option (“proleader” distortion) and distortion to weaken a trailing option (“antitrailer” distortion). To address this, recent research introduced new response scales to SEP. We explore whether and how these new response scales might influence the very proleader and antitrailer processes that they were designed to capture (“reactivity”). We used the SEP method with concurrent verbal reporting: fifty family physicians verbalized their thoughts as they evaluated patient symptoms and signs (“cues”) in relation to two competing diagnostic hypotheses. Twenty-five physicians evaluated each cue using the response scale traditional to SEP (a single response scale, returning a single measure of distortion); the other twenty-five did so using the response scales introduced in recent studies (two separate response scales, returning two separate measures of distortion: proleader and antitrailer). We measured proleader and antitrailer processes in verbalizations, and compared verbalizations in the single-scale and separate-scales groups. Response scales did not appear to affect proleader processes: the two groups of physicians were equally likely to bolster their leading diagnosis verbally. Response scales did, however, appear to affect antitrailer processes: the two groups denigrated their trailing diagnosis verbally to differing degrees. Our findings suggest that the response scales used to measure information distortion might influence its constituent processes, limiting their generalizability across and beyond experimental studies.

## Introduction

Decision makers are not unbiased in their treatment of newly arriving evidence. Rather, they appear to evaluate incoming information in a way that supports an emerging hypothesis or preference [1–22]. This phenomenon is known as “predecisional information distortion” (hereafter “distortion”) [1, 2] and it implies that reasoning is bidirectional: processed information feeds into conclusions, but emerging conclusions in turn shape the manner in which new information is processed [3, 7]. This is thought to aid decision makers in attaining and maintaining a state of “cognitive coherence” [3, 7, 23, 24]; that is, a consistent representation of the choice options and their attributes.

Distortion is pervasive (for reviews, see [25, 26]). It manifests in a variety of populations (e.g., [4, 15, 27]) and choice domains (e.g., [15, 18, 27]). It affects the evaluation of real and hypothetical options [28], and that of neutral and diagnostic information [2, 15]. It occurs when preferences are installed experimentally and when they are allowed to develop naturally [1, 2], and it occurs regardless of whether a final choice among options is expected [2, 5]. It has been linked to predecisional commitment to an option [15] and eventual selection of an option [13, 18, 20]. Indeed, it has been linked to the selection of an inferior option [10] and appears to withstand monetary incentives for accuracy [29, 30].

While distortion has been studied extensively, the response scales used to measure it have not. Distortion is most often measured by the “stepwise evolution of preference” (SEP) method [2]. By this method, participants typically face a choice between two options (e.g., restaurants). Items of information (“cues”) describing features of the two options are presented sequentially (e.g., menus, opening hours). In response to each cue, participants are asked to rate the extent to which it favors one option over the other (e.g., a Visual Analogue Scale (VAS) anchored at “favors option A” and “favors option B”). They are also asked to indicate their preferred option, based on all information seen so far. Distortion is thought to occur when a cue is rated as overly favorable toward the option that is preferred at the time. This is usually measured relative to the responses provided by a separate control group: a group who rate the same cues in relation to the same two options, but who are precluded from developing any preferred option that might bias their ratings.

Recently, the limitations of this procedure were pointed out by three different teams of researchers [19–21]. The response scale is comparative: it measures distortion as the relative advantage afforded a leading option over its competitor. Therefore, it cannot measure separately the two processes thought to comprise distortion: distortion to strengthen a leading option (“proleader” distortion) and distortion to weaken a trailing option (“antitrailer” distortion). To overcome this, Blanchard and colleagues [19], DeKay and colleagues [20] and Nurek and colleagues [21] introduced new response scales to SEP. They replaced the single, comparative response scale (e.g., “favors option A” to “favors option B”) with separate, option-specific response scales (e.g., “no support” to “strong support” for option A; “no support” to “strong support” for option B [21]). This allowed for 1) separate evaluation of information in relation to leading and trailing options, and therefore 2) separate measurement of proleader and antitrailer distortion. Two of these studies found reliable evidence for proleader distortion [19, 20] and all three found reliable evidence for antitrailer distortion.

Response scales are a powerful task feature. A change in response mode can impact findings in important ways. For example, Lichtenstein and Slovic [31] identified preference reversals when participants were required to choose vs. price monetary gambles (*A* and *B*): participants tended to select *A* but priced *B* higher. Hsee [32] identified similar reversals when participants were required to price consumer items one-at-a-time vs. side-by-side: separate pricing favored *A* but simultaneous pricing favored *B*. In the current context, the simple provision of separate response scales for leading and trailing options could provide the opportunity–and indeed, alter the tendency–to distort each option. For example, separate evaluation of cues in relation to leading and trailing options might force participants to consider support for the trailing option more fully than they would otherwise have done (“consider the opposite” [33]). Equally, if separate response scales call attention to the trailing option, they might also call for its denigration. In short, the response scales used to measure distortion (single vs. separate) might influence the type of distortion observed (proleader vs. antitrailer) (“reactivity” [34, 35]).

We aimed to investigate the effect of response scales on proleader and antitrailer processes. We achieved this using SEP with concurrent verbal reporting: we asked family physicians to verbalize their thoughts as they evaluated sequentially presented cues (items of patient data) in relation to two competing diagnostic hypotheses, in the context of two clinical cases. One group of physicians did so using the single response scale traditional to SEP (the “single-scale” group); another group did so using the separate response scales introduced in more recent work (the “separate-scales” group). We measured proleader and antitrailer processes in verbalizations, and compared these verbalizations in the single-scale vs. separate-scales groups. The findings contribute to a topical body of work concerning the measurement of proleader and antitrailer processes in predecisional information distortion.

## Materials and Methods

### Ethics statement

Ethical approval for this study was obtained from King’s College London Biomedical Sciences, Dentistry, Medicine and Natural and Mathematical Sciences Research Ethics Subcommittee (ref BDM/13/14-104). Informed consent was obtained from participants in writing.

### Materials

We employed two clinical cases, which were used by Kostopoulou and colleagues [15] and Nurek and colleagues [21] to investigate predecisional information distortion in medical diagnosis. One described a patient with dyspnea (which could be due to either heart failure or chronic lung disease) and the other a patient with fatigue (which could be caused by either diabetes or depression). Each patient case began with a brief introduction, which contained the patient’s name, age, sex, health complaint and a “diagnostic steer”; i.e., three clinical cues that provided strong support for one of the two competing diagnoses. For each patient case, half of the physicians saw a steer favoring diagnosis A and half saw a steer favoring diagnosis B (randomly assigned and counterbalanced). Thereafter, each patient case delivered a sequence of 4–5 “neutral” clinical cues. Each neutral cue provided some support for both diagnostic hypotheses, and equal support for the two. Materials took the form of questionnaires, constructed and administered online using Qualtrics.

### Procedure

We arranged telephone interviews with the participating physicians. Ten minutes prior to the interview, physicians received an e-mail containing a link to the study website and a telephone number to dial toll-free from a landline. Physicians were aware that telephone calls would be audio-recorded. Once on the phone with the researcher (MN), physicians began the online questionnaire. They were instructed to think aloud, i.e., verbalize any thoughts that came to mind, as they completed the questionnaire. They were asked not to explain their reasoning, but simply to report the contents of working memory [34, 36–39]. They were also asked to read aloud anything that appeared on the screen, i.e., cues and questions. To ensure that physicians understood these instructions, the questionnaire began with a non-clinical practice task. This allowed physicians to grow accustomed to thinking aloud and gave the researcher an opportunity to provide feedback [34, 36, 38, 40].

**Fig 1:**
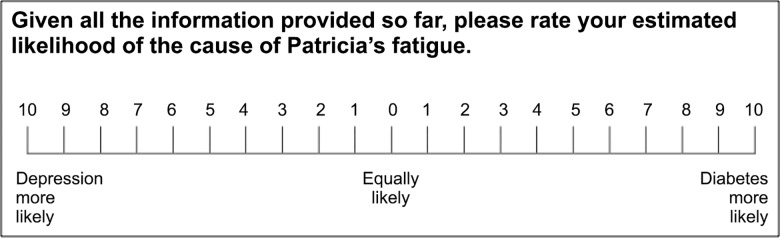
Scale used to estimate diagnostic likelihood after 1) the steer and 2) each cue evaluation. The same scale was used by Kostopoulou et al. [15] and Nurek et al. [21].

Physicians then encountered the two patient cases in a random order. Each case began with a patient introduction, which contained a diagnostic steer (see *Materials*). Based on this, physicians gave an initial estimate of diagnostic likelihood on a 21-point VAS, anchored at “diagnosis A more likely” and “diagnosis B more likely” (Fig 1).

Physicians then encountered four (dyspnea case) or five (fatigue case) neutral cues, which were presented sequentially and in a random order. They were asked to respond to each cue, providing 1) a rating of its diagnostic value and 2) an updated estimate of diagnostic likelihood, based on all the information seen so far.

**Fig 2:**
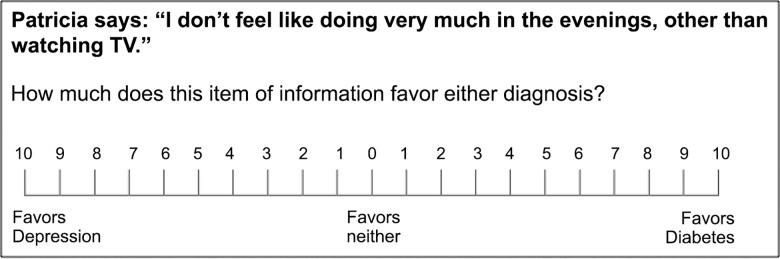
Scale used to collect ratings of cue diagnosticity in the single-scale group. The same scale was used by Kostopoulou et al. [15]. Participants were required to place one mark upon the scale.

**Fig 3:**
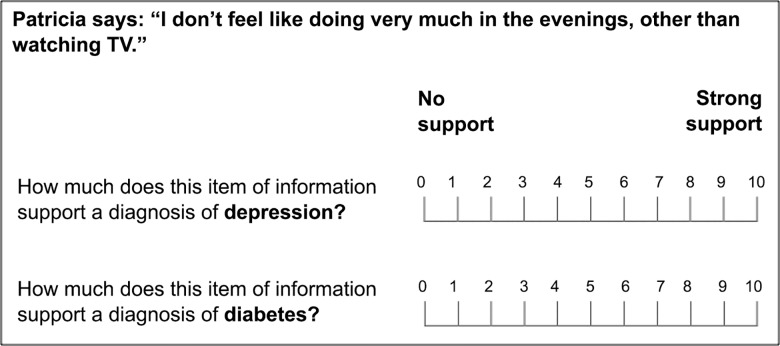
Scales used to collect ratings of cue diagnosticity in the separate-scales group. The same scales were used by Nurek et al. [21]. Participants were required to place one mark upon each scale. The diagnosis evaluated first was counterbalanced across participants.

Ratings of diagnostic value were cast under one of two response modes. Half of the sample (the single-scale group) was randomly assigned to rate the diagnostic value of each cue using a single 21-point VAS, anchored at “favors diagnosis A” and “favors diagnosis B” (Fig 2). This single response scale–traditional to SEP–was used by Kostopoulou et al. [15] to measure distortion in physicians’ diagnostic judgments. The other half of the sample (the separate-scales group) rated the diagnostic value of each cue using two separate 11-point VASs, one per diagnostic hypothesis, each anchored at “no support” and “strong support” (Fig 3). These separate response scales–new to SEP–were used by Nurek et al. [21] to measure proleader and antitrailer distortion in physicians’ diagnostic judgments. In both groups, estimates of diagnostic likelihood were always cast on a 21-point VAS (Fig 1).

If physicians fell silent during the questionnaire, the researcher said in a neutral voice “keep talking” [36, 38]. Upon completion of the two cases, the telephone call was terminated. The questionnaire concluded with a Debrief Sheet, which explained the nature of the bias under study (information distortion) and gave physicians an opportunity to withdraw their data (tick-box).

### Data analysis

#### Measuring distortion in cue ratings

By the SEP method, the distortion of a cue is measured relative to its “baseline” rating, i.e., the mean rating given thereto by a separate control group of participants [2]. Control participants typically evaluate exactly the same cues as those seen by the experimental group, but they are precluded from developing any overarching preference (or leading diagnosis) that could bias their cue ratings [2, 15, 21].

Baseline ratings for the present cues were readily available from our previous work. In the study by Kostopoulou et al. [15], a control group of 36 physicians provided baseline ratings for these cues using a single cue evaluation scale (Fig 2). In the study by Nurek et al. [21] (study 1), a control group of 43 physicians provided baseline ratings for the same cues using two separate cue evaluation scales (Fig 3). The procedure used to obtain baseline ratings was described at length in these publications [15, 21] and is also explained here in the Supporting Information (S1 Text).

We used the baseline cue ratings gathered by Kostopoulou et al. [15] to calculate “leader-signed distortion” [2] in the single-scale group, and the baseline cue ratings gathered by Nurek et al. [21] (study 1) to calculate leader-signed proleader and antitrailer distortion in the separate-scales group. Distortion was calculated exactly as described by Kostopoulou et al. [15] and Nurek et al. [21], respectively. Full details are available in the Supporting Information (S2 Text).

#### Coding verbal protocols

Recorded interviews were transcribed and analyzed by the first author. The second author analyzed a randomly selected subset (20%) for the purposes of measuring agreement.

Protocol analysis aimed to identify and characterize, per cue, verbalizations about the diagnosis that was leading at the time (“Verb_Lead”) and verbalizations about the diagnosis that was trailing at the time (“Verb_Trail”).

For each cue, Verb_Lead was defined as any utterance/s connecting the given cue to the diagnosis that was leading at the time. Likewise, Verb_Trail was defined as any utterance/s connecting the given cue to the diagnosis that was trailing at the time. We excluded 1) utterances made in the absence of a leading diagnosis (i.e., when the most recent estimate of diagnostic likelihood was 0 = “equally likely”) and 2) utterances made when physicians were providing estimates of diagnostic likelihood (Fig 1) rather than cue evaluations (Figs 2 or 3).

We developed a coding scheme to characterize the Verb_Lead and Verb_Trail per cue. Each Verb (Lead/Trail) received one of four possible codes: “supportive”, “non-supportive”, “unclear” or “nothing”. Definitions and examples for each category appear in Table 1.

**Table 1:** Coding scheme for protocol analysis.

Code	Definition	Examples
**Supportive**	The cue was perceived to support the diagnosis	1. Cue is considered a feature of the diagnosis:“ • [cue] is a presenting factor for [diagnosis A]” • “you can get [cue] with [diagnosis A]” • “[cue] is consistent with [diagnosis A]” 2. Cue is perceived to increase the likelihood of the diagnosis: • “[cue] makes [diagnosis A] more likely” • “[cue] would add to the diagnosis of [diagnosis A]” • “[cue] suggests/supports/favors [diagnosis A]” 3. Cue leads participant towards the diagnosis: • “given [cue], I’m now thinking [diagnosis A]” • “[cue] sends me towards [diagnosis A]” • “[cue] makes me want to explore [diagnosis A]” • “[cue] makes me consider [diagnosis A] as a possibility”
**Non-supportive**	The cue was not perceived to support the diagnosis	1. Cue is not considered a feature of the diagnosis: • “[cue] is not a presenting factor for [diagnosis A]” • “You don’t usually see [cue] with [diagnosis A]” • “[cue] is not consistent with [diagnosis A]” 2. Cue is not perceived to increase the likelihood of the diagnosis:* • “[cue] doesn’t help me to know whether this is [diagnosis A]” • “[cue] tells me nothing about [diagnosis A]” • “[cue] doesn’t add anything to [diagnosis A]” • “[cue] is irrelevant to/has no bearing on [diagnosis A]” • “[cue] doesn’t suggest/support/favor [diagnosis A] 3. ”Cue leads participants away from the diagnosis: • “[cue] doesn’t make me think of [diagnosis A]” • “[cue] moves me a bit away from [diagnosis A]” • “I wouldn’t be looking for [cue] with [diagnosis A]”
**Unclear**	The cue was evaluated in relation to the diagnosis, but perceived support for the diagnosis was unclear	1. Cue’s support for the diagnosis is ambiguous: • “[cue] might be because of [diagnosis A] and it might not be because of [diagnosis A]” • “[cue] doesn’t differentiate between [diagnosis A] and [diagnosis B]” 2. Cue’s support for the diagnosis is questioned rather than stated: • “could [cue] suggest [diagnosis A]?”
**Nothing**	The cue was not evaluated in relation to the diagnosis	N/A

Each participant was assigned two codes per cue: one in relation to the diagnosis that was leading at the time (“Verb_Lead”) and one in relation to the diagnosis that was trailing at the time (“Verb_Trail”).

* Note: many of the utterances in this subcategory suggest that a cue is irrelevant to a diagnosis: the cue is not perceived to support the diagnosis but it is not perceived to negate it either. Such utterances could be coded as “unclear”. We categorized them as such in a second coding of the data and our findings did not change. Full details are available in the Supporting Information (S3 Text: point 8).

The four categories were mutually exclusive and exhaustive. If a given Verb (e.g., Verb_Lead) contained conflicting utterances (e.g., some supportive utterances and some non-supportive utterances), we coded the physician’s final utterance. Coders were blinded to the ratings that each physician provided for each cue (unless the physician verbalized this), and to the extent of each cue’s distortion. They were aware only of the physician’s leading diagnosis at the time, which was integral to the physicians’ utterances and used to identify Verb_Lead and Verb_Trail. Full guidelines for coding verbal protocols are available in the Supporting Information (S3 Text).

#### Measuring proleader and antitrailer processes in verbalizations (separate-scales group)

Separate scales for the evaluation of two competing options allows for the separate measurement of proleader and antitrailer distortion in cue ratings. Therefore, we used data from the separate-scales group to explore the relationship between distortion in relation to an option (leading/trailing) and verbalizations about that option. We hypothesized that:

*H*_*1*_: As proleader distortion increases, so will the tendency to make “supportive” verbalizations about the leading diagnosis.

*H*_*2*_: As antitrailer distortion increases, so will the tendency to make “non-supportive” verbalizations about the trailing diagnosis.

To test these hypotheses, we ran two multilevel logistic regression models (one per hypothesis), with random intercepts to account for cue ratings clustered within physicians. The model for *H*_*1*_ used a physician’s proleader distortion score for a cue to predict his/her Verb_Lead for that cue (1 = supportive; 0 = any other category). The model for *H*_*2*_ used a physician’s antitrailer distortion score for a cue to predict his/her Verb_Trail for that cue (1 = non-supportive; 0 = any other category).

#### Assessing the effect of response scales on verbalizations (single-scale vs. separate-scales groups)

If *H*_*1*_ and *H*_*2*_ are true, then we have developed a valid means of measuring proleader and antitrailer processes in verbalizations rather than cue ratings. We can then explore the effect of response scales (single vs. separate) on these verbalizations. We hypothesized that:

*H*_*3*_: If response scales affect proleader processing, then the single-scale group and the separate-scales group will differ in their tendency to make “supportive” verbalizations about the leading diagnosis.

*H*_*4*_: If response scales affect antitrailer processing, then the two groups will differ in their tendency to make “non-supportive” verbalizations about the trailing diagnosis.

We note that there are many ways in which response scales might affect proleader and antitrailer processes. For example, if separate (vs. single) response scales raise the profile of the trailing diagnosis (see Introduction), then this could threaten the superiority of the leader by activating concepts inconsistent with it. This might 1) incite proleader and/or antitrailer distortion to restore cognitive coherence, or it might 2) inhibit proleader and/or antitrailer distortion by interrupting cognitive coherence (“consider the opposite”). Furthermore, these are not mutually exclusive: separate (vs. single) response scales could, for example, inhibit proleader distortion but exacerbate antitrailer distortion. Thus our hypotheses are non-directional; we aimed to assess whether response scales might influence proleader and antitrailer processes in any way.

To test these hypotheses, we ran two multilevel logistic regression models (one per hypothesis), each with a random intercept. The model for *H*_*3*_ used a physician’s response scale (1 = separate, 0 = single) to predict his/her Verb_Lead per cue (1 = supportive; 0 = any other category). The model for *H*_*4*_ used a physician’s response scale (1 = separate, 0 = single) to predict his/her Verb_Trail per cue (1 = non-supportive; 0 = any other category).

### Recruitment and sample

From a database of UK family physicians who had taken part in previous studies by the second author, we invited 287 via e-mail. We did not invite physicians who had participated in our previous studies of distortion because the same patient cases were used [15, 21]. We also made use of social media, advertising the study online in reputable networking groups exclusive to UK family physicians. We informed physicians that participation would involve a ±20 minute telephone call with the researcher (audio-recorded), where they would be required to verbalize their thoughts as they reasoned over two fictitious patient cases (accessible online). They would receive a £20 Amazon e-voucher upon completion of data collection.

Of the 287 physicians e-mailed, 44 responded (15%), 27 participated (9%) and one was excluded because the response scales did not display accurately on his/her computer screen. This is a low response rate compared to our previous studies of distortion (e.g., [21]: study 1 = 48%, study 2 = 49%). This is likely due to the fact that invited physicians were required to agree on a specific time for participation and could not participate at their convenience like in the previous studies. Furthermore, the time commitment was larger than in the previous studies (20 rather than 10 minutes). Physicians may also have found the prospect of thinking aloud while solving clinical cases–and being audiotaped–daunting or uncomfortable.

A further 24 physicians were recruited via social media (where a response rate cannot be calculated), yielding a final sample of 50 physicians: 52% females, 27 to 62 years of age (*M* = 38.5, *SD* = 7.9), with 0 to 34 years of experience in family medicine (*M* = 8.3, *SD* = 8.1). The single-scale (*n* = 25) and separate-scales (*n* = 25) groups were similar in age (*M* for single = 37.9, *SD* = 7.5; *M* for separate = 39.1, *SD* = 8.4), experience (*M* for single = 6.8, *SD* = 7.0; *M* for separate = 9.7, *SD* = 9.0), gender (single = 52% female; separate = 52% female) and recruitment method (single = 52% social media; separate = 44% social media).

For completeness, we report demographic details for the physicians who provided baseline ratings of cues, used to calculate information distortion in previous studies [15, 21] and in the present one. Kostopoulou et al. [15] obtained baseline data from 36 physicians: 46% female, 26 to 64 years of age (*M* = 47.2, *SD* = 11.6), with 0 to 39 years in family medicine (*M* = 17.1, *SD* = 11.1). Nurek et al. [21] (study 1) obtained baseline data from 43 physicians: 56% female, 29 to 61 years of age (*M* = 39.1, *SD* = 8.9), with 0 to 34 years in family medicine (*M* = 9.9, *SD* = 9.9).

## Results

### Distortion in cue ratings

In the single-scale group, distortion was averaged across cues per physician. The grand mean for distortion was 1.24 ([0.52, 1.96], *SD* = 1.74, *t* (24) = 3.55, *p* = 0.002, d = 0.71). In the separate-scales group, proleader and antitrailer distortion were each averaged across cues per physician. The grand mean for proleader distortion was 0.71 ([-0.02, 1.44], *SD* = 1.77, *t* (24) = 2.00, *p* = 0.057, d = 0.40) and that for antitrailer distortion was 0.63 ([-0.02, 1.27], *SD* = 1.56, *t* (24) = 2.01, *p* = 0.055, d = 0.40).

We compared distortion to that identified in our previous studies [15, 21]. Kostopoulou et al. [15] and Nurek et al. [21] employed three patient cases rather than two, one of which featured three diagnostic (i.e., non-neutral) cues at its end. The additional patient case and the diagnostic cues were excluded from the present study to minimize the cognitive and temporal load placed upon our participants, who were thinking aloud. We thus recalculated distortion in the previous studies, limiting the data to the two patient cases and neutral cues employed here. Findings are presented in Table 2. Distortion appeared consistent in the present (column 1) and previous (column 2) studies, returning no significant differences (column 3). Proleader and antitrailer distortion did not differ reliably from one another, in the present study (mean difference = 0.08 [-1.09, 1.25], *t* (24) = 0.14, *p* = 0.889, d = 0.03) or in Nurek et al.’s previous study (reanalyzed) (mean difference = 0.35 [-0.12, 0.83], *t* (95) = 1.47, *p* = 0.146, d = 0.15).

**Table 2:** Mean distortion in the present study vs. previous studies (Kostopoulou et al., [15] and Nurek et al. [21], study 1).

Distortion	Present study^a^	Previous studies^b^	Mean difference
**Single-scale**	1.24	1.38	-0.14
	[0.52, 1.96]	[1.00, 1.76]	[-0.99, 0.71]
	*t*(24) = 3.55, *p* = 0.002	*t*(101) = 7.12, *p*< 0.001	*t*(125) = -0.33, *p* = 0.740
	d = 0.71	d = 0.70	d = 0.07
**Separate-scales: Proleader**	0.71	0.34	0.37
	[-0.02, 1.44]	[0.06, 0.61]	[-0.27, 1.02]
	*t*(24) = 2.00, *p* = 0.057	*t*(95) = 2.42, *p* = 0.017	*t*(119) = 1.14, *p* = 0.255
	d = 0.40	d = 0.25	d = 0.26
**Separate-scales: Antitrailer**	0.63	0.69	-0.06
	[-0.02, 1.27]	[0.43, 0.95]	[-0.66, 0.54]
	*t*(24) = 2.01, *p* = 0.055	*t*(95) = 5.23, *p*< 0.001	*t*(119) = -0.19, *p* = 0.848
	d = 0.40	d = 0.53	d = 0.04

^*a*^ *n* present study = 25 (single-scale) and 25 (separate-scales)

^b^ *n* previous studies = 102 (single-scale; [15]) and 96 (separate-scales; [21], study 1).

### Inter-rater agreement for verbalizations

Inter-rater agreement was substantial for verbalizations about the leading diagnosis (*kappa* for Verb_Lead = 0.84, *p* < 0.001) and for verbalizations about the trailing diagnosis (*kappa* for Verb_Trail *=* 0.85, *p* < 0.001).

### Measuring proleader and antitrailer processes in verbalizations (separate-scales group)

We hypothesized that, in the separate-scales group, proleader distortion would be associated with supportive verbalizations about the leading diagnosis (*H*_*1*_). Table 3 conveys this group’s verbalizations about the leader, separately for the cues that featured proleader distortion (proleader distortion > 0, column 1) and the cues that did not (proleader distortion ≤ 0, column 2). Supportive verbalizations appeared more common when proleader distortion was present (78%, 101/129) than when it was absent (50%, 38/76). Per cue, greater proleader distortion (continuous) was associated with greater odds of a supportive Verb_Lead (OR = 1.28 [1.06, 1.55], *p =* 0.010).

**Table 3:** Separate-scales group: frequency and proportion of codes assigned to verbalizations about the leading diagnosis (Verb_Lead).

Verb_Lead	Cues featuring proleader distortion	Cues featuring no proleader distortion	Total
**Supportive**	101 (78%)	38 (50%)	139 (68%)
**Non-supportive**	6 (5%)	23 (30%)	29 (14%)
**Unclear**	12 (9%)	12 (16%)	24 (12%)
**Nothing**	10 (8%)	3 (4%)	13 (6%)
**Total**	**129**	**76**	**205**^**a**^

^a^ The separate-scales group evaluated 225 cues in total (9 per physician). Eighteen cues were excluded, as the physicians in question held no leading diagnosis at the time that these cues were evaluated (diagnostic likelihood = 0). Two cues were not verbally evaluated due to technical problems.

We also hypothesized that, in the separate-scales group, antitrailer distortion would be associated with non-supportive verbalizations about the trailing diagnosis (*H*_*2*_). Table 4 conveys this group’s verbalizations about the trailer, separately for the cues that featured antitrailer distortion (antitrailer distortion > 0, column 1) and the cues that did not (antitrailer distortion ≤ 0, column 2). Non-supportive verbalizations appeared more common when antitrailer distortion was present (38%, 45/119) than when it was absent (9%, 8/86). Per cue, greater antitrailer distortion (continuous) was associated with greater odds of a non-supportive Verb_Trail (OR = 1.44 [1.21, 1.70], *p* < 0.001).

**Table 4:** Separate-scales group: frequency and proportion of codes assigned to verbalizations about the trailing diagnosis (Verb_Trail).

Verb_Trail	Cues featuring antitrailer distortion	Cues featuring no antitrailer distortion	Total
**Supportive**	51 (43%)	61 (71%)	112 (55%)
**Non-supportive**	45 (38%)	8 (9%)	53 (26%)
**Unclear**	16 (13%)	8 (9%)	24 (12%)
**Nothing**	7 (6%)	9 (11%)	16 (8%)
**Total**	**119**	**86**	**205**^**a**^

^a^ The separate-scales group evaluated 225 cues in total (9 per physician). Eighteen cues were excluded, as the physicians in question held no leading diagnosis at the time that these cues were evaluated (diagnostic likelihood = 0). Two cues were not verbally evaluated due to technical problems.

For completeness, we ran the same analyses in regards to the single-scale group. Per cue, greater distortion (continuous) was associated with greater likelihood of both a supportive Verb_Lead (OR = 1.20 [1.11, 1.30], *p* < 0.001) and a non-supportive Verb_Trail (OR = 1.21 [1.07, 1.37], *p* = 0.003).

### Assessing the effect of response scales on verbalizations (single-scale vs. separate-scales groups)

We hypothesized that if response scales affect proleader processing, then the single-scale group and the separate-scales group should differ in their tendency to provide supportive verbalizations with respect to the leading diagnosis (*H*_*3*_). Table 5 conveys verbalizations about the leading diagnosis, separately for the single-scale group (column 1) and the separate-scales group (column 2). The two groups did not appear to differ in their tendency to provide supportive Verb_Leads (single = 64%, 134/210; separate = 68%,139/205) and our multilevel logistic regression confirmed this (OR = 1.20 [0.77, 1.90], *p* = 0.421).

**Table 5:** Frequency and proportion of codes assigned to verbalizations about the leading diagnosis (Verb_Lead).

Verb_Lead	All cues: single-scale	All cues: separate-scales	Total
**Supportive**	134 (64%)	139 (68%)	273 (66%)
**Non-supportive**	7 (3%)	29 (14%)	36 (9%)
**Unclear**	44 (21%)	24 (12%)	68 (16%)
**Nothing**	25 (12%)	13 (6%)	38 (9%)
**Total**	**210**^**a**^	**205**^**a**^	**415**

^a^ Each group evaluated 225 cues in total (9 per physician). Thirty-two cues were excluded (single-scale = 14; separate-scales = 18), as the physicians in question held no leading diagnosis at the time that these cues were evaluated (diagnostic likelihood = 0). Three cues were not verbally evaluated due to technical problems (single-scale = 1; separate-scales = 2).

We also hypothesized that if response scales affect antitrailer processing, then the two response-scale groups should differ in their tendency to provide non-supportive verbalizations with respect to the trailing diagnosis (*H*_*4*_). Table 6 conveys verbalizations about the trailing diagnosis, separately for the single-scale group (column 1) and the separate-scales group (column 2). The two groups appeared to differ in their tendency to provide non-supportive Verb_Trails (single = 7%, 14/210; separate = 26%, 53/205) and our multilevel logistic regression confirmed this (OR = 4.86 [2.16, 10.94], *p* < 0.001): the separate-scales group was significantly more likely than the single-scale group to make non-supportive verbalizations about their trailing diagnosis.

**Table 6:** Frequency and proportion of codes assigned to verbalizations about the trailing diagnosis (Verb_Trail).

Verb_Trail	All cues: single-scale	All cues: separate-scales	Total
**Supportive**	114 (54%)	112 (55%)	226 (55%)
**Non-supportive**	14 (7%)	53 (26%)	67 (16%)
**Unclear**	43 (20%)	24 (12%)	67 (16%)
**Nothing**	39 (19%)	16 (8%)	55 (13%)
**Total**	**210**^**a**^	**205**^**a**^	**415**

^a^ Each group evaluated 225 cues in total (9 per physician). Thirty-two cues were excluded (single-scale = 14; separate-scales = 18), as the physicians in question held no leading diagnosis at the time that these cues were evaluated (diagnostic likelihood = 0). Three cues were not verbally evaluated due to technical problems (single-scale = 1; separate-scales = 2).

Notably, the separate-scales group also appeared more likely to make non-supportive verbalizations about their leader (Table 5: single = 3%, 7/210; separate = 14%, 29/205). We explored this in a multilevel logistic regression (random intercept) that used response scale (+0.5 = separate, -0.5 = single), diagnosis (+0.5 = trailing, -0.5 = leading) and their interaction to predict Verb (1 = non-supportive, 0 = any other category) per cue. It returned a reliable effect for response scale (OR = 4.77 [2.49, 9.14], *p* < 0.001), a reliable effect for diagnosis (OR = 2.09 [1.23, 3.57], *p* = 0.007) and no reliable interaction (OR = 1.03 [0.35, 2.98], *p* = 0.963). This suggests that the separate (vs. single) scale group was more likely to make non-supportive verbalizations about both the leader and the trailer, with no significant difference between the two.

Finally, we compared the two groups in terms of attention paid to the leader vs. the trailer. We used response scale (+0.5 = separate, -0.5 = single), diagnosis (+0.5 = trailing, -0.5 = leading) and their interaction to predict Verb per cue: Verb = 0 if no evaluation was verbalized (“nothing”) and Verb = 1 if any evaluation was verbalized (any other category). We found a reliable effect for response scale (OR = 2.39 [1.15, 4.95], *p* = 0.020), no reliable effect for diagnosis (OR = 0.69 [0.42, 1.12], *p* = 0.130) and no reliable interaction (OR = 1.35 [0.51, 3.56], *p* = 0.548). This suggests that the separate (vs. single) scale group was more likely to attend to both the leader and the trailer, with no significant difference between the two.

## Discussion

We explored the effect of two different response scales on the processes thought to underlie predecisional information distortion. Family physicians thought aloud as they evaluated clinical cues in relation to two competing diagnostic hypotheses, in the context of two patient cases. One group did so using the single response scale traditional to SEP (the single-scale group); the other group did so using the separate response scales introduced more recently (the separate-scales group). We measured proleader and antitrailer processes in verbalizations, and compared these verbalizations in the two study groups.

### Verbal measurement of proleader and antitrailer processing

In the separate-scales group, we identified a correspondence between cue distortion in relation to a diagnostic hypothesis (leading/trailing) and verbalizations about that diagnostic hypothesis. As expected, proleader distortion was associated with supportive verbalizations about the leader and antitrailer distortion was associated with non-supportive verbalizations about the trailer. In the single-scale group, similar patterns were identified: cue distortion was associated with both the former and the latter.

These findings advance the literature in two ways. Firstly, they suggest that verbalizations can serve as a valid indicator of proleader and antitrailer processes. Our study is not the first to measure biased predecisional processing in verbalizations (e.g., [41–43]), but it is–to our knowledge–the first to validate its verbal measure against an established behavioral one; that is, information distortion as measured by numerical cue ratings (SEP). Secondly, our findings suggest that even when distortion is measured on a single response scale, both proleader and antitrailer processes operate to some extent. Our study is not the first to suggest this [19], but it is–to our knowledge–the first to provide evidence based on data collected using the single scale itself.

### Effect of response scales on proleader and antitrailer processing

We compared the frequency of proleader and antitrailer verbalizations across the single-scale and separate-scales groups. Response scales did not appear to affect proleader processing: the two groups were equally likely to verbalize support for their leading diagnosis. The scales did, however, appear to affect antitrailer processing: the separate-scales group was significantly more likely to denigrate their trailing diagnosis. One possible explanation is attentional: separate (vs. single) response scales raised the profile of the trailing diagnosis, perhaps explaining its denigration.

Interestingly, the leading diagnosis also received more attention and more non-supportive verbalizations in the separate-scales group than in the single-scale group. Asking physicians to evaluate cues in relation to each diagnostic competitor may have led them to question their leading hypothesis, simply by activating concepts inconsistent with it (“consider the opposite” [33]). Alternatively, separate (vs. single) response scales might simply be more conducive to negative evaluations of a cue’s support for a diagnosis, given that their lowest point (0) is anchored at “no support” (vs. “favors neither”).

Our findings suggest that the response scales used to measure distortion might influence its constituent processes, which could limit generalizability across and beyond experimental studies. Distortion as measured on separate response scales might not reflect distortion as measured on a single one, and both might misrepresent distortion as it occurs in practice. In the present study, physicians were required to 1) evaluate incoming items of patient data in relation to competing diagnostic hypotheses and 2) update their diagnostic belief after each. Faced with a new item of patient information, physicians in practice might do neither.

Nevertheless, using a more ecologically valid design that involved Active Information Search [44] and no intermediary rating of cues, Kostopoulou, Mousoulis, and Delaney [45] found evidence for both proleader and antitrailer processes in physicians’ diagnostic reasoning. Furthermore, the in/exclusion of intermediary cue ratings does not appear to affect final choices [9, 18, 46]). If indeed distortion operates in daily diagnosis and to negative end, our findings suggest that debiasing is possible. To the extent that task features such as response scales affect evaluative processes, then these processes are malleable (i.e., subject to manipulation). Future work could explore whether and how different approaches to cue evaluation might mitigate distortion. DeKay and colleagues [20] identified a near-significant (*p* = 0.063) decrease in distortion when they altered the wording of their separate response scales: distortion (averaged across proleader and antitrailer) was lower when the separate scales encouraged absolute evaluation of an option (“very unappealing” to “very appealing” for option A [B]) rather than relative evaluation of an option (“strongly disfavors” to “strongly favors” for option A [B]). Rewording the separate scales did not eliminate proleader or antitrailer distortion (nor alter their relative magnitudes) [20], but its potential to perhaps reduce distortion is encouraging. If a debiasing approach to cue evaluation is identified, then distortion could perhaps be countered through metacognitive training (“metacognitive strategies” [47]; “cognitive forcing” [48]), though questions surround the feasibility and long-term efficacy of such training in medical practice [49–51].

### Limitations

The think aloud methodology carries inherent limitations. Arguably, introspective access to cognitive processes is limited [52]: participants can only verbalize cognitions that are heeded or “focally attended” [36, 39]. To the extent that relevant cognitions occur automatically or pre-attentively [53, 54], verbal reports may be incomplete [36, 39].

A second limitation of the think aloud method is its potential for interference with the task of interest [34]. Asking physicians to think aloud while solving a diagnostic problem could alter their reasoning in a number of ways, e.g., through competition for cognitive resources for the diagnostic vs. the verbalization task, through improved recall via auditory feedback, or through the generation of new inferences and improved strategies on account of heightened reflection [34]. Furthermore, asking physicians to think aloud in the (telephonic) presence of a researcher could induce or exacerbate “social desirability” bias [55], which might further alter responses. We note also that our control participants did not think aloud; they simply provided cue ratings online. To the extent that concurrent verbalization influenced cue ratings, this procedural difference between the experimental and the control groups could threaten our estimates of distortion. However, a prominent and comprehensive review found no evidence to suggest that thinking aloud alters thought processes: on objective measures of task performance, participants in think-aloud conditions did not differ from their “silent control” counterparts [37] (see also [36], chapter 2). In a recent study, family physicians diagnosed patient cases under both think aloud (via telephone) and silent conditions; diagnostic accuracy did not differ between the two [56]. Likewise, the distortion identified in the present study did not differ from that identified in our previous studies [15, 21] (Table 2), where physicians completed the same tasks under silent conditions. (For a more detailed discussion of distortion in our present vs. previous studies, see S4 Text).

We note that our sample size was small, due to a low response rate. This may have limited our quantitative analysis, which must temper our broader conclusions. Future work might apply the present methods to larger samples drawn from more accessible populations. We note also that verbal reports are rich; a more fine-grained analysis than ours could generate new or deeper insights into a complex phenomenon. For example, future work might explore subtypes of “supportive” and “non-supportive” verbalizations, sorted by (e.g.) strength or kind. Alternatively, future work could obtain a direct measure of verbal distortion–akin to SEP’s direct measure of distortion in cue ratings–by collecting verbal data under control conditions (that is, while participants provide baseline ratings of cues, see S1 Text) and comparing these to verbal data collected under experimental conditions. By this nuanced measure, verbal proleader [antitrailer] distortion would require a more supportive [less supportive] verbalization than was provided at baseline.

Finally, protocol analysis requires reflexivity. Coders were aware of the study hypotheses. They were also aware of each physician’s leading diagnosis at each cue evaluation, which could not be extricated from cue verbalizations themselves. This may have created a meta-bias: coders may have been biased to identify bias in physicians’ verbalizations (e.g., supportive [non-supportive] verbalizations about leading [trailing] diagnoses). However, findings from *H*_*1*_ and *H*_*2*_ suggest not: coders were blinded to the distortion displayed per cue, yet coding appeared to reflect this well in both response-scale groups.

Despite these limitations, the present study lends insight into the processes underlying predecisional information distortion, and the factors that may affect them. It contributes to this fresh and dynamic literature both methodologically and theoretically. Firstly, it triangulates numeric ratings with verbal data: it maps distortion as measured by response scales to verbalizations as measured by think aloud, and uses this mapping to explore reactivity in the response scales themselves. This mixed-methods approach may be extended and refined in future work. Secondly, it identifies reactivity in the response scales used to measure distortion. This could impact the generalizability of findings between and beyond experimental studies, which should be considered when designing and reporting future work.

## Supporting Information

S1 Dataset (CSV)

S1 Text Procedure for obtaining baseline ratings for clinical cues in previous studies of information distortion in physicians’ diagnostic judgments. (PDF)

S2 Text Calculation of leader-signed distortion in the single-scale group and the separate-scales group. (PDF)

S3 Text Guidelines for coding verbal protocols. (PDF)

S4 Text Distortion in our present vs. previous studies. (PDF)

S5 Text Data key: Variables in “S1 Dataset”. (PDF)
