# Supplementary material for: What You Find Depends on How You Measure It: Reactivity of Response Scales Measuring Predecisional Information Distortion in Medical Diagnosis
Source: PLoS One. 2016 Sep 14;11(9):e0162562. doi: 10.1371/journal.pone.0162562 (PMC5023159; doi:10.1371/journal.pone.0162562)
Supplement: S1 Text — (PDF) [file pone.0162562.s002.pdf]

S1 Text. Procedure for obtaining baseline ratings for clinical cues in previous studies of information distortion in physicians' diagnostic judgments.

Kostopoulou et al. [15] and Nurek et al. [21] obtained baseline ratings for clinical cues as follows. Firstly, cues were not presented in the context of coherent patient scenarios; rather, the cues comprising the different scenarios were collected and scrambled (i.e., presented as a randomly-ordered list). Each cue was presented in relation to a brand new patient, who was introduced by a letter rather than a name (e.g., Patient C), a health complaint (e.g., fatigue) and minimal demographic information (sex and age). To reduce the likeness of patients who presented with the same health complaint, patient age was varied by ≤ 4 years above or below the age that was stated in the corresponding patient scenario. Decoy cues were also included, which were unrelated to the patient scenarios; instead, they pertained to entirely different areas of medicine (e.g., gastroenterology). Physicians were asked to evaluate each cue in relation to two diagnostic hypotheses (depression and diabetes for fatigue cues; heart failure and chronic lung disease for dyspnea cues), using either a single response scale (in the study by Kostopoulou et al. [15], Fig 2) or two separate response scales (in the study by Nurek et al. [21], Fig 3). Given that each cue pertained to a brand new patient, physicians were never asked to provide cumulative estimates of diagnostic likelihood (Fig 1).
