# Supplementary material for: What You Find Depends on How You Measure It: Reactivity of Response Scales Measuring Predecisional Information Distortion in Medical Diagnosis
Source: PLoS One. 2016 Sep 14;11(9):e0162562. doi: 10.1371/journal.pone.0162562 (PMC5023159; doi:10.1371/journal.pone.0162562)
Supplement: S2 Text — (PDF) [file pone.0162562.s003.pdf]

S2 Text. Calculation of leader-signed distortion in the single-scale group and the separate-scales group.

We calculated leader-signed distortion in the single-scale group in the manner described by Kostopoulou et al. [15] (p. 834). Specifically, each physician in the experimental group provided one rating per cue (Fig 2). To calculate its distortion, we computed the difference between the experimental physician's rating of the cue and the mean baseline rating for that cue (derived from Kostopoulou et al. [15]). This difference was then signed in accord with the experimental physician's leading diagnosis at the time: positive if it favored the leading diagnosis and negative if it did not. If a physician held no leading diagnosis at the time of the cue's evaluation – i.e., if the most recent estimate of diagnostic likelihood was 0 ("equally likely") – then distortion was not calculated on this cue.

We calculated leader-signed proleader and antitrailer distortion in the separate-scales group in the manner described by Nurek et al. [21] (study 1, p. 575). Specifically, each physician in the experimental group provided two ratings per cue, one for each diagnostic hypothesis (Fig 3). To compute distortion in relation to the diagnosis that was leading at the time (proleader distortion), we calculated the difference between the experimental physician's rating of the cue in relation to the leading diagnosis and the mean baseline rating of the same cue in relation to the same diagnosis (derived from Nurek et al. [21], study 1). If the experimental rating was *higher* than the mean baseline rating, this difference was signed positive; if not, it was signed negative. To compute distortion in relation to the diagnosis that was trailing at the time (antitrailer distortion), we calculated the difference between the experimental physician's rating of the cue in relation to the trailing diagnosis and the mean baseline rating of the same cue in relation to the same diagnosis (derived from Nurek et al. [21], study 1). If the experimental rating was *lower* than the mean

baseline rating, this difference was signed positive; if not, it was signed negative. Therefore, each physician received two distortion scores per cue (proleader and antitrailer) and positive scores always indicated distortion in the expected direction: strengthening [weakening] the leading [trailing] diagnosis. Again, distortion was not calculated in the absence of a leading diagnosis (i.e., when the preceding estimate of diagnostic likelihood was 0 (“equally likely”)).
