# Supplementary material for: What You Find Depends on How You Measure It: Reactivity of Response Scales Measuring Predecisional Information Distortion in Medical Diagnosis
Source: PLoS One. 2016 Sep 14;11(9):e0162562. doi: 10.1371/journal.pone.0162562 (PMC5023159; doi:10.1371/journal.pone.0162562)
Supplement: S3 Text — (PDF) [file pone.0162562.s004.pdf]

S3 Text. Guidelines for coding verbal protocols.

1. **Per cue, we identify the Verb_Lead and the Verb_Trail.**

   Verb_Lead = any utterance (or group of utterances) connecting the presenting cue to the leading diagnostic hypothesis.

   Verb_Trail = any utterance (or group of utterances) connecting the presenting cue to the trailing diagnostic hypothesis.

2. **We ignore the following utterances:**

   a) Utterances made when a participant has no leading diagnosis, i.e. when his/her previous rating of diagnostic likelihood is 0 = "equally likely". These utterances are not coded and they do not influence coding.

   b) Utterances made when participants are estimating diagnostic likelihood (Fig 1). These utterances are not coded and they do not influence coding. We code only utterances made when participants are providing cue evaluations (Figs 2 and 3).

3. **Each Verb (Lead/Trail) can be assigned only one of the four possible codes: supportive, non-supportive, unclear or nothing.**

   If a Verb contains conflicting utterances, i.e. some utterances suggesting that support is present and some utterances suggesting that support is absent, we code the participant's final utterance (i.e., his/her own conclusion).

4. **Coding relative judgments**

   (e.g., diagnosis A > diagnosis B; diagnosis A = diagnosis B)

   In relative judgments, a cue's support for a diagnosis is expressed in a relative way rather than an absolute way. We identify four types of relative judgment, which we code as follows.

a) diagnosis A > diagnosis B

Example: “This item supports/favors diagnosis A more than diagnosis B”.

Description: Cue is perceived to support diagnosis A more than diagnosis B, but absolute support for diagnosis B is unclear.

Coding: Verb_A = supportive. Verb_B = unclear, unless there are other utterances in the cue’s evaluation suggesting that support for B is present (or absent), in which case Verb_B is coded as supportive (or non-supportive).

b) diagnosis A, ¬diagnosis B

Example: “This item supports/favors diagnosis A rather than diagnosis B”.

Description: Cue is perceived to support diagnosis A to the exclusion of diagnosis B.

Coding: Verb_A = supportive, Verb_B = non-supportive.

c) diagnosis A > ?

Example: “This item supports/favors diagnosis A more”.

Description: Cue is perceived to support diagnosis A “more”, but diagnosis B is not mentioned.

Coding: Verb_A = supportive, Verb_B = nothing.

d) diagnosis A = diagnosis B

Example: “This item doesn’t support one diagnosis over the other”; “This item is not particularly discriminating”; “This item favors neither diagnosis”.

Description: Cue is perceived to support diagnoses A and B equally, with no information as to how much support it provides for either/both.

Coding: Verb_A = unclear, unless there are other utterances in the cue’s evaluation suggesting that support for A is present (or absent), in which case Verb_A is coded as supportive (or non-supportive). Likewise, Verb_B = unclear, unless there are other

utterances in the cue's evaluation suggesting that support for B is present (or absent), in which case Verb_B is coded as supportive (or non-supportive).

5. **Coding statements of low support**

Statements of low support suggest some, but very little, support for a diagnosis. It can thus be difficult to assess whether the spirit of the evaluation is supportive or non-supportive. To do so, we differentiate between statements of low support that are framed positively vs. negatively.

a) Low support framed positively

Example: "This item favors diagnosis A, but not very much", "This item supports diagnosis A a little bit".

Description: Cue is perceived to provide low support for diagnosis A, and this support is framed positively.

Coding: Verb_A = supportive.

b) Low support framed negatively

Example: "This doesn't really support diagnosis A", "This doesn't support diagnosis A much", "This doesn't particularly favor diagnosis A".

Description: Cue is perceived to provide low support for diagnosis A, and this support is framed negatively.

Coding: Verb_A = non-supportive, unless there are other utterances in the cue's evaluation that suggest support for A. If such utterances are present (e.g., "This doesn't support diagnosis A much. Maybe a little bit."), then Verb_A = supportive. If not (e.g., "This doesn't support diagnosis A much."), then Verb_A = non-supportive.

6. **Coding numeric expressions**

Utterances stating the numeric rating/s provided for a cue were ignored; they were

"crossed out" of participants' transcripts and they were not coded. Surrounding text was coded as above.

Example 1: "This item favors diagnosis A, I think. ~~I'll give it a 4 favoring diagnosis A~~".

Coding: Verb_A = supportive, Verb_B = nothing.

Example 2: "This item strongly supports diagnosis A. ~~I'll give diagnosis A a 9 and diagnosis B a 2~~".

Coding: Verb_A = supportive, Verb_B = nothing.

Utterances *describing* the numeric rating/s provided for a cue (e.g., "high", "low") were coded.

Example: "Support for diagnosis A? I'd rate that as high, ~~maybe an 8~~. And support for diagnosis B? Very low, ~~maybe a 1~~".

Coding: Verb_A = supportive, Verb_B = non-supportive.

7. **References to previous items**

Participants are asked to evaluate each new item on its own. However, they do sometimes refer to previous items when encountering a new one. In coding such statements, we differentiate between a) references to previous information that inform the evaluation of the current cue (which are coded) and b) references to previous information rather than the current cue (which are ignored).

a) References to previous information that inform evaluation of the current cue

Example: "Given [previously seen item/s], I would say that this item favors diagnosis A and not diagnosis B".

Description: Participant incorporates previous information into his/her evaluation of the presenting cue.

Coding: Verb_A = supportive, Verb_B = non-supportive.

b) References to previous information, with no evaluation of the current cue

Example: "I'm still thinking about [previously seen item/s], which strongly suggest diagnosis A rather than diagnosis B".

Description: Participant addresses previous information, and does not address the current cue.

Coding: Verb_A = nothing, Verb_B = nothing.

8. A second coding of the data

Participants occasionally make statements suggesting that a cue is irrelevant to a diagnosis (see Table 1 in the main text). The cue is not perceived to support the diagnosis (thus the code is "non-supportive") but we note that the cue is not perceived to negate the diagnosis either. In a second coding of the data, we classified all such utterances as "unclear" rather than "non-supportive".

Relevant findings (H_2 and H_4) did not change:

H_2: in the separate-scales group, greater antitrailer distortion of a cue was associated with greater likelihood of a non-supportive Verb_Trail for that cue (OR = 1.42 [1.23, 1.64], $p < 0.001$). Likewise, in the single-scale group, greater distortion of a cue was associated with greater likelihood of a non-supportive Verb_Trail for that cue (OR = 1.25 [1.14, 1.36], $p < 0.001$).

H_4: the separate-scales group was significantly more likely than the single-scale group to provide a non-supportive Verb_Trail (OR = 4.52 [2.08, 9.85], $p < 0.001$).

For the interested reader: we reclassified 3 out of 14 (21%) non-supportive Verb_Trails in the single-scale group and 12 out of 53 (23%) non-supportive Verb_Trails in the separate-scales group. In the latter group, 45 of the 53 had featured antitrailer distortion and 10 of these (22%) were reclassified; 8 of the 53 had featured no antitrailer distortion and 2 of these (25%) were reclassified.
