# Supplementary material for: What You Find Depends on How You Measure It: Reactivity of Response Scales Measuring Predecisional Information Distortion in Medical Diagnosis
Source: PLoS One. 2016 Sep 14;11(9):e0162562. doi: 10.1371/journal.pone.0162562 (PMC5023159; doi:10.1371/journal.pone.0162562)
Supplement: S4 Text — (PDF) [file pone.0162562.s005.pdf]

S4 Text. Distortion in our present vs. previous studies.

Though distortion was comparable in our present and previous studies, two points of departure are noteworthy. Firstly, we recalculated distortion in the previous studies to reflect the cues used in the present one. Our reanalysis returned findings consistent with those reported in the previous studies, with one exception: proleader distortion was reliable in our reanalysis (*M* = 0.34 [0.06, 0.61], *p* = 0.017) but not in Nurek et al.'s original analysis (*M* = 0.20 [−0.07, 0.47], *p* = 0.138; [21], study 1, p. 576). The original analysis included diagnostic cues, which might receive less proleader distortion than neutral cues (mean difference = 0.32 [-0.04, 0.68], *p* = 0.082; [21], study 1, p. 576). The original analysis also included an additional patient case, in which mean proleader distortion was rather low (*M* = 0.10 [-0.29, 0.48], *p* = 0.619; [21], study 1, unpublished data).

Secondly, we note that Nurek et al.'s data (once reanalyzed) returned reliable evidence for proleader and antitrailer distortion (Table 2, column 2). In the present study, the significance of each was marginal (Table 2, column 1). However, 95% confidence intervals overlapped considerably: the present intervals encompassed and extended those of Nurek et al. (Table 2). Wider intervals suggest increased vulnerability to noise, likely due to the much smaller sample employed presently (*n* = 25) than previously (*n* = 96). Noise may have been due to random fluctuations or non-random ones: Nurek et al. [21] identified individual differences in physicians' "preferred" mode of distortion (proleader vs. antitrailer), which would indeed exacerbate noise in small samples.
