# Supplementary material for: What You Find Depends on How You Measure It: Reactivity of Response Scales Measuring Predecisional Information Distortion in Medical Diagnosis
Source: PLoS One. 2016 Sep 14;11(9):e0162562. doi: 10.1371/journal.pone.0162562 (PMC5023159; doi:10.1371/journal.pone.0162562)
Supplement: S5 Text — (PDF) [file pone.0162562.s006.pdf]

S5 Text. Data key: Variables in "S1_Dataset.csv"

Primary variables:

**Physician**	A physician identifier
**Group**	Response-scale group<br>• 0: single-scale<br>• 1: separate-scales
**Scenario_Order**	• 1 = scenario seen first<br>• 2 = scenario seen second
**Scenario**	• 0: dyspnea<br>• 1: fatigue
**Cue_Order**	Order in which cues were presented<br>• 1 = first cue<br>• 2 = second cue, etc.
**Proleader**	A physician's proleader distortion score for a given cue:<br>• single-scale: a physician's distortion of a given cue (identical to antitrailer)<br>• separate-scales: a physician's distortion of a given cue in relation to the leading diagnosis
**Antitrailer**	A physician's antitrailer distortion score for a given cue:<br>• single-scale: a physician's distortion of a given cue (identical to proleader)<br>• separate-scales: a physician's distortion of a given cue in relation to the trailing diagnosis
**Verb_Lead**	A physician's verbal evaluation of a cue in relation to the leading diagnosis:<br>• 0: nothing<br>• 1: unclear<br>• 2: supportive<br>• 3: non-supportive
**Verb_Trail**	A physician's verbal evaluation of a cue in relation to the trailing diagnosis:<br>• 0: nothing<br>• 1: unclear

	• 2: supportive<br>• 3: non-supportive

The remaining variables are derived from "Verb_Lead" and "Verb_Trail":

**Verb_Lead_Nothing**	Indicates whether a physician evaluated a cue in relation to the leading diagnosis:<br>• 0: nothing<br>• 1: other (unclear, supportive or non-supportive)
**Verb_Trail_Nothing**	Indicates whether a physician evaluated a cue in relation to the trailing diagnosis:<br>• 0: nothing<br>• 1: other (unclear, supportive or non-supportive)
**Verb_Lead_Supportive**	Indicates whether a physician's verbal evaluation of a cue in relation to the leading diagnosis was supportive:<br>• 0: other (nothing, unclear or non-supportive)<br>• 1: supportive
**Verb_Trail_Nonsupportive**	Indicates whether a physician's verbal evaluation of a cue in relation to the trailing diagnosis was non-supportive:<br>• 0: other (nothing, unclear or supportive)<br>• 1: non-supportive
